# Access to mental health under Medicare has stalled – What now?

**DOI:** 10.1177/00048674251396025

**Published:** 2025-12-11

**Authors:** Sebastian Rosenberg, Ian Hickie

**Affiliations:** 1 Brain and Mind Centre, The University of Sydney, Sydney, NSW, Australia

**Keywords:** Mental health services, equity, accountability, reform

## Abstract

One of the key concerns of recent national mental health policy has been to lift the rate of population access to mental health services.

The origin of this concern can be found in successive findings from the National Survey of Mental Health and Wellbeing (Australian Bureau of Statistics, 1998). The first time this survey was conducted, in 1997, it found 38% of Australians with a 12-month mental health disorder received care for their condition. Despite considerable subsequent attention to policies, services and funding, the second Survey, undertaken in 2007, found the rate of community access to mental health care had declined a little to 35% (Slade et al., 2009).

One of the most significant policy changes made at this time was the Council of Australian Governments (CoAG) National Action Plan on Mental Health (Council of Australian Governments, 2006). With eventual spending of more than $5bn over 5 years, CoAG’s plan provided an umbrella for all Australian governments to increase their investment in mental health (Rosenberg et al., 2012). By far the largest component of the Federal Government’s contribution to the Action Plan was the establishment of the Better Access Program, a series of new billable items, permitting subsidised public access to psychology services under the Medicare Benefits Schedule.

This journal recently reported that the impact of this Program (among other things) has been to lift the rate of public access to mental health care to 47%, ‘a meaningful overall increase’ from 2007 (Harris et al., 2025). The increase they noted was further supported by the findings of the National Mental Health Commission’s 2024 Report Card (as being 45.1%).

This boost would seem welcome news, particularly given almost $30 m is paid in benefits for Medicare-subsidised mental health-specific services every week (Australian Institute of Health and Welfare, 2025).

However, in making the comparison with the 2007 Survey findings, the Harris article did not refer to a 2013 paper by Whiteford et al. (2013) which indicated the rate of access to care had apparently already reached 46% by 2009–2010.

In other words, it is possible to suggest there has been no ‘meaningful’ increase in public access to mental health services for the past 15 years.

Figure 1 presents the mental health services provided under Medicare by profession, since the Better Access Program began. Initial increases in service provision are evident for psychologists and general practitioners (GPs), with rates peaking in around 2018. For all professions, the rate of services provided per 100,000 population has subsequently declined.

**Figure 1.**
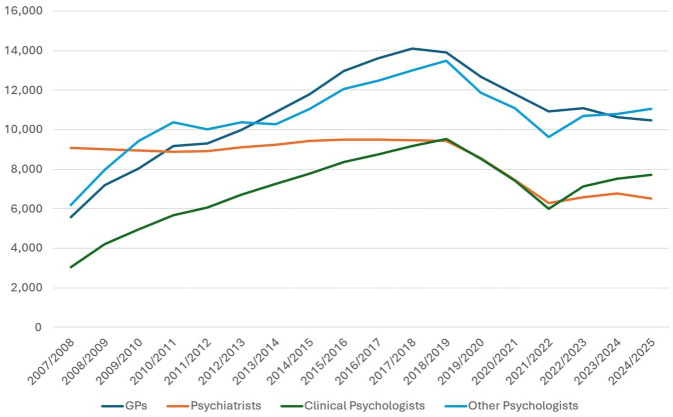
Medicare Mental Health Services Provided per 100,000 population.

For psychologists, one reason may be their more recent capacity to provide uncapped sessions of capacity-building support, or ‘functional recovery’ services, under the National Disability Insurance Scheme, as opposed to the capped ‘clinical recovery’ sessions which exist under Medicare. No data on psychology spending under the NDIS is available to verify this.

Medicare data indicate that in 2024–2025 there were about 1.3 m GP mental health care treatment plans written. Of these, just over half a million were reviewed, or just over 40%. GPs may choose to use or record patient interactions using a variety of Medicare Item numbers. However, based on this data anyway, assessment of the impact of the GP Mental Health Care Plans is the exception, not the rule. Patient evaluation and monitoring is at best doubtful.

It is also worth noting that the Productivity Commission’s Report on Government Services (ROGS) found that new mental health clients treated under Medicare accounted for 34.5% of all clients in 2014–2015 (the first year this was recorded). In the most recent year, 2023–2024, this figure was 25.8%.

So not only has the overall rate of community access to mental health care not risen but also increasingly services are also being provided to the same clients, year on year.

This raises key questions about service targeting and quality, bearing in mind the original intent of the Better Access Program was to help people with mild-to-moderate mental health needs (Whiteford, 2019).

In lieu of more appropriate care, and as found in the last evaluation (Australian Government, 2022), Program services are no doubt being provided to people with more severe needs than originally envisaged. People are not getting the help they need.

Given that access rates have stalled and the proportion of repeat customers continues to increase, there is an urgent need for an overhaul of the way we respond to mental illness.

This overhaul should go beyond Medicare services to include the pre-dominantly hospital-based mental health services provided by state and territory governments. Other ROGS data also shows that the rate of new clients into these services remains unchanged over the past decade, at just over 41%.

Clients seem stuck in models of mental health service provision are still based largely on 20th century notions of place, and workforce-constrained deployment of primary, secondary and tertiary levels of care, that themselves are often poorly coordinated and inequitable in distribution (Meadows et al., 2015). Over the last 30 years, these models have demonstrated limited capacity to drive national mental health reform, let alone at the scale required now.

Increasing access to mental health care matters. Staying below 50%, especially when demand or incidence is increasing, simply will not deliver the population level outcomes we need.

Rather than tinker with obsolete service models, a key focus for the next round of mental health reform should be delivery on the long-promised community mental health service which was supposed to underpin deinstitutionalisation. Intended to be a feature of reform, community mental health services have actually dwindled. Getting specialist community mental health care, clinical or psychosocial, is very rare across Australia (Rosenberg and Harvey, 2021).

A vibrant, multidisciplinary community mental health sector could be the focus and bedrock of the next national mental health agreements, prioritising prevention, early intervention and hospital avoidance, helping states and territories address the overwhelming pressures they currently face in their emergency departments and acute psychiatric wards. It would also be consistent with recommendations made by the Productivity Commission (2025), which found the existing national agreement not fit for purpose.

To enhance the quality of care delivered, and achieve genuine population-level outcomes (Skinner et al., 2022), we seriously need to deploy radically new approaches to the equitable delivery of well coordinated services. We can already draw on new modelling approaches to guide planning and development of these services (Hosseini et al., 2025).

This kind of guided, equitable investment, at scale, can lead towards systemic stability rather than the current crisis (Skinner et al., 2023). There are already examples of the regional application of this smarter approach, helping decision-makers optimise the limited mental health resources they have available (Crosland et al., 2024).

Existing models of mental health service provision have demonstrated the limit of their capacity to drive national mental health reform at the scale required. We can use new tools for better planning and system design, leading to better, more equitable access to mental health care.
